# Editorial: Video-assisted surgery in oncology

**DOI:** 10.3389/fonc.2024.1420249

**Published:** 2024-07-10

**Authors:** Jianrong Zhang, He Liu, Jinbo Chen, Zhiming Ma, Long Jiang

**Affiliations:** ^1^ Melbourne Medical School & Centre for Cancer Research, Faculty of Medicine, Dentistry and Health Sciences, University of Melbourne, Melbourne, VIC, Australia; ^2^ Victorian Comprehensive Cancer Centre, Melbourne, VIC, Australia; ^3^ Department of Orthopedics, The Second Hospital of Jilin University, Changchun, China; ^4^ Department of Urology, Xiangya Hospital, Central South University, Changsha, China; ^5^ Department of Gastrointestinal Nutrition and Hernia Surgery, The Second Hospital of Jilin University, Changchun, China; ^6^ Department of Thoracic Surgery/Oncology, Guangzhou Institute of Respiratory Disease, China State Key Laboratory of Respiratory Disease, National Clinical Research Center for Respiratory Disease, First Affiliated Hospital of Guangzhou Medical University, Guangzhou, China

**Keywords:** neoplasms, surgical oncology, video-assisted surgery, minimally invasive surgical procedure, endoscopy

Video-assisted surgery (VAS) has emerged as a safe and effective approach to the treatment of diseases, including cancer. In contrast to open surgery, which is the traditional type of surgery, VAS allows resection to be minimally invasive for improved peri- and post-operative patient outcomes. Moreover, VAS can potentially enhance the effectiveness of healthcare service utilization (1). This Research Topic, “Video-Assisted Surgery in Oncology”, aims to provide a transdisciplinary forum with rigorous evidence on the use of VAS in cancer.

Research comparing patient outcomes between VAS and open surgery, robotic-assisted surgery (RAS) and radiotherapy is a common focus. Two studies among the publications focus on the comparison between RAS and VAS. Specifically, a study by Zeng et al. in non-small-cell lung cancer (NSCLC) after neoadjuvant immunochemotherapy yielded positive results for RAS in terms of the conversion rate to open surgery (thoracotomy), number of lymph nodes (LNs) and the stations harvested, post-operative pain score, and importantly, higher pathological N1 and N2 staging. Another study, by Fu et al., revolved around the upper third of gastric cancer, with positive RAS results in the number of LNs and quality of life assessments within one year post-operatively. For this comparison, we observed the superiority of RAS mostly in operation experience, peri-operative and short-term post-operative outcomes in eligible patients. Its superiority in long-term outcomes needs to be confirmed by more studies. In observational studies, the analysis may consider how patient demographics, especially socioeconomic status, may interact with the comparison, given the high cost of RAS.

Similar conditions in the above peri-operative and post-operative outcomes were found in sleeve lobectomy via VAS and RAS combined versus thoracotomy for centrally located lung cancer, as reported by Chen et al. Of note, sleeve lobectomy for this type of cancer is a technically demanding procedure, especially via RAS and VAS, so comparable results on survival should be acceptable.

New and advanced VAS techniques are included in this Research Topic. In pre-operative settings, Zhang et al. reported the feasibility of applying three-dimensional computed tomography-bronchography and angiography (3D-CTBA) for basal segmentectomy via VAS to treat lung neoplasms, with the advantage of clearly displaying the anatomical structure of the bronchi, pulmonary arteries and veins for accurate resection. Wang et al. explored the safety and effectiveness of pre-operative (versus peri-operative) injection of a tracer agent carbon nanoparticles (CNs) to protect the parathyroid glands in patients receiving VAS for papillary thyroid cancer. In addition to precise surgery, better applications in imaging and biomarker testing are also needed for accurate staging and post-operative surveillance, particularly with positron emission tomography (PET)-CT scans, whole genome sequencing, and liquid biopsy.

In the peri-operative setting, a randomized controlled trial (RCT) by Fan et al. investigating opioid-free anesthesia (OFA) with esketamine versus opioid anesthesia in spontaneously ventilated video-assisted thoracic surgery (SV-VATS) for NSCLC, found 1) equivalent pain control within 24 hours post-operatively, 2) superior circulatory and respiratory stability, quality of pulmonary collapse but 3) a longer time to consciousness with higher doses of propofol and dexmetomidine. With respect to OFA, important research questions to be answered are the post-discharge outcomes. For example, longer-term pain control and adverse events, in addition to its association with opioid over prescribing, prolonged use, diversion, and human harm.

Therapeutic strategies with new indications are suggested in the following publications. Shen et al. proposed 4L LN dissection for left-sided NSCLC, especially lung adenocarcinoma, given the station metastasis with a prevalence of 25% and medium-term prognostic impact. Gao et al. explored the safety and feasibility of laparoscopic transduodenal ampullectomy (LTDA) in 9 cases with pre-malignant tumors of the ampulla of Vater (AoV). Wang et al. introduced a novel “zero-line” incision design in VAS gasless unilateral trans-axillary approach (GUA) thyroidectomy for thyroid cancer.

The state of the art also includes the extension of VAS indications to more advanced cancers with neoadjuvant or adjuvant treatment. We encourage studies aimed at improving the efficacy, postoperative safety and tolerability of neoadjuvant/adjuvant treatment. Comparing the neoadjuvant/adjuvant treatment regimens can be a promising option (2). In addition, we should point out the peri-operative adjuvant strategy with VAS and the use of VAS for cancer treatment regimens. Recent advances include hyperthermic intrathoracic chemotherapy (HITHOC) with VATS for malignant pleural mesothelioma (3) and laparoscopic hyperthermic intraperitoneal chemotherapy (HIPEC) for gastric cancer with peritoneal metastasis (4).

With many advances in VAS and further innovations to come, the implementation question should be how to scale utilization to benefit more patients. Understanding the learning curve is fundamental. Huang et al. found that 44 operations are needed to achieve acceptable perioperative outcomes for subsegmental resection via uniportal VATS for patients with early-stage lung cancer, including operative time, intraoperative bleeding, and length of hospital stay. Also, in uniportal VATS for early-stage lung cancer, Song et al. found reviewing recorded surgical videos to be beneficial for learning lobectomy, suggesting that 53 operations are necessary to achieve proficiency based on the above outcomes as well as complications and lymph nodes harvested. The next step is continuing medical education. It may need to be improved as differences in patient prognostic outcomes and hospital utilization may be impacted by surgeons and hospitals, even within developed countries. For example, better results have been found in high-volume surgeons and hospitals (5, 6).

Inequitable access is a central issue. Advanced VAS techniques have been well adopted in high-performance settings and regions. However, VAS is still poorly applied in low- and middle-income countries, mainly due to limited infrastructural capacity and clinical experience (7). VAS capacity and its studies in the countries must be further developed to meet the increasing demand due to the growing cancer burden (8). As VAS becomes the standard of care in cancer surgery, affordable VAS should be a goal, regardless of geographic or socioeconomic conditions. Along with feasibility and efficacy studies, efforts also need to be made to conduct cost-effectiveness studies and implementation studies on geographic and socioeconomic disparities.

Finally, the study of the timeliness to VAS in oncology is insufficient but essential (9), as delays in cancer care are still common worldwide (10). With the more frequent use of VAS with other cancer treatment, especially for more advanced cancer, studies investigating the optimal timing of the subsequent treatment are also warranted (9). As a transdisciplinary forum, this Research Topic celebrates the current advances and innovations in VAS and calls for more evidence to support its implementation.

## Author contributions

JZ: Writing – original draft, Writing – review & editing. HL: Writing – review & editing. JC: Writing – review & editing. ZM: Writing – review & editing. LJ: Writing – review & editing.
